# Expression and Functions of Galectin-7 in Human and Murine Melanomas

**DOI:** 10.1371/journal.pone.0063307

**Published:** 2013-05-03

**Authors:** Katherine Biron-Pain, Andrée-Anne Grosset, Françoise Poirier, Louis Gaboury, Yves St-Pierre

**Affiliations:** 1 INRS-Institut Armand-Frappier, Laval, Québec, Canada; 2 Institut Jacques Monod, CNRS, UMR 7592, Univ Paris Diderot, Sorbonne Paris Cité, Paris, France; 3 Institut de Recherche en Immunologie et Cancérologie, Montreal, Québec, Canada; Faculdade de Medicina, Universidade de São Paulo, Brazil

## Abstract

The identification of galectin-7 as a p53-induced gene and its ability to induce apoptosis in many cell types support the hypothesis that galectin-7 has strong antitumor activity. This has been well documented in colon cancer. However, in some cases, such as breast cancer and lymphoma, its high expression level correlates with aggressive subtypes of cancer, suggesting that galectin-7 may have a dual role in cancer progression. In fact, in breast cancer, overexpression of galectin-7 alone is sufficient to promote metastasis to the bone and lung. In the present work, we investigated the expression and function of galectin-7 in melanoma. An analysis of datasets obtained from whole-genome profiling of human melanoma tissues revealed that galectin-7 mRNA was detected in more than 90% of biopsies of patients with nevi while its expression was more rarely found in biopsies collected from patients with malignant melanoma. This frequency, however, was likely due to the presence of normal epidermis tissues in biopsies, as shown our studies at the protein level by immunohistochemical analysis. Using the experimental melanoma B16F1 cell line, we found that melanoma cells can express galectin-7 at the primary tumor site and in lung metastasis. Moreover, we found that overexpression of galectin-7 increased the resistance of melanoma cells to apoptosis while inducing *de novo egr-1* expression. Overexpression of galectin-7, however, was insufficient to modulate the growth of tumors induced by the subcutaneous injection of B16F1 cells. It also failed to modulate the dissemination of B16F1 cells to the lung.

## Introduction

Melanoma accounts for 4% of dermatological cancers but is responsible for 80% of mortalities related to skin cancer [1]. In addition, its incidence is increasing at a higher rate than other cancer types. Most melanomas are resistant to chemotherapy and immunotherapy, most likely as a result of resistance to apoptosis [2], [3]. Systemic treatments include the administration of nonspecific immune-stimulating cytokines, immunization with cancer cells or molecules, adoptive T cell transfer, small inhibitors of melanoma oncogenes and blocking antibodies against inhibitory immune molecules, like Ipilimumab [4]. Therefore, it is of great interest to identify new, relevant biological targets to discover new therapeutics against melanoma.

Galectins are a family of 15 animal lectins with a unique carbohydrate recognition domain that binds to β-galactoside derivatives [5], [6]. They can have intracellular (cytoplasmic and/or nuclear) or extracellular functions, even without a signal sequence, which is essential for the classical secretory pathway [7], [8]. Galectins function during embryonic development, wound healing, apoptosis, protein trafficking, intercellular adhesion, cell migration, immune responses and cancer [9]–[11]. Galectin-1 and galectin-3 are the most well studied members of the galectin family, but evidence has shown that other galectins are also important and have specific expression patterns. This is true for galectin-7, which was initially described as a marker for keratinocytes [12], [13]. In normal tissues, the expression of galectin-7 is normally confined to stratified epithelia [14]. In epithelial cancer, however, its expression is often significantly altered and may have distinct implications. In a model of human colon carcinoma, for instance, the exogenous expression of galectin-7 aids in eliminating tumor cells through its pro-apoptotic function [15]. This connection between galectin-7 and apoptosis is supported by studies showing that galectin-7 is induced in human colon cancer cells following activation of the p53 pathway [16]. Galectin-7 is also associated with the sensitivity of human cervical carcinoma cells to apoptosis induced by chemotherapeutic agents [17]. However, galectin-7 has been associated with cancer progression in chemically induced models of rat mammary carcinoma [18] and human hypopharyngeal squamous cell carcinoma [19]. Moreover, galectin-7 overexpression in murine lymphoma and breast cancer cells has been shown to increase their ability to metastasize [20]–[22]. This dual role in controlling tumor growth is not unusual for members of the galectin family; for example, it has been well documented for galectin-3 [9], [23]. In the present work, we have investigated the expression pattern of galectin-7 in melanoma and used a well-characterized melanoma model to study its functional relevance.

## Materials and Methods

### Mice

Breeder pairs for a C57BL/6 mouse colony were purchased from Jackson Laboratory (Bar Harbor, ME). Galectin-7-deficient mice (KOG7) in a C57BL/6 background have been described previously [24]. Male and female mice were bred in our animal facility and maintained under specific pathogen–free conditions in accordance with institutional guidelines. All animal studies were approved by the Institutional Animal Care and Use Committee (CISAU) of the INRS-Institut Armand-Frappier.

### Cell Lines and Reagents

The mouse melanoma B16F1 cell line was obtained from the American Type Culture Collection (ATCC). The aggressive variant B16F10 cell line was a generous gift from Dr. Alain Lamarre (INRS-Institut Armand-Frappier) [25]. The human melanoma cell lines (888mel, 537mel, SK23 and Mel-FB) were a generous gift from Dr. Réjean Lapointe (Research Centre, Centre Hospitalier de l’Université de Montréal (CRCHUM)) [26]. The mouse thymic lymphoma line 164T2 and its aggressive variant S19 have been previously described [27]. All cell lines were maintained in RPMI 1640 complete medium supplemented with 8% (v/v) FCS, 2 mM _L_-glutamine, 10 mM Hepes buffer, 1 mM sodium pyruvate and 0.075% sodium bicarbonate. All cell culture products were purchased from Life Technologies (Burlington, ON, Canada). Anti-cleaved PARP-1 antibody was purchased from Epitomics (Burlingame, CA); anti-β-actin antibody was purchased from Sigma-Aldrich (St. Louis, MO); anti-EGR-1 antibody was purchased from Santa Cruz (CA, USA); anti-human galectin-7 monoclonal antibody was purchased from R & D Systems; methyl [^3^H]thymidine was purchased from Perkin Elmer (Waltham, MA); cell culture lysis reagent (CCLR) and passive lysis buffer were purchased from Promega (Madison, WI); RIPA lysis buffer was purchased from Thermo Scientific (Rockford, USA); and buffered formaldehyde solution was purchased from Fisher Scientific (Toronto, ON). All other reagents were purchased from Sigma-Aldrich unless otherwise indicated.

### RNA Isolation and Semiquantitative PCR

Total RNA was isolated from tissues using Trizol reagent according to the manufacturer’s instructions (Invitrogen Canada, Inc., Burlington, ON). Briefly, total RNA (2 µg) was reverse transcribed using Omniscript reverse transcriptase (Qiagen, Mississauga, ON) and PCR amplified using the following conditions: 94°C for 0.5 min, 58°C for galectin-7 and β-actin or 60°C for *Egr-1* for 1 min, and 72°C for 1 min. Then, a final extension step was performed at 72°C for 10 min. The primers used for PCR amplification were (5′-CCATGTCTGCT-ACCCATCAC-3′; in exon 2) for sense murine galectin-7 and (5′-GCTTAGAA-GATATTCAATGAATGC-3′; in exon 5) for antisense; (5′-TAATAGCAGCAGCAGCA-CCAGC-3′) for sense murine EGR-1 and (5′-GTCGTTTGGCTGGGATAACTCG-3′) for antisense, and (5′-CATGGATGACGATAT-CGCTGCGC-3′) for sense β-actin and (5′-GCTGTCGCCACGCTCGGTCAGGAT-3′) for antisense. Amplification was carried out in a thermal cycler (model PTC-100, MJ Research, Watertown, MA) using equal amounts of RNA that was reverse transcribed and amplified by PCR for 35 cycles with gene-specific primers. Each amplification step was performed in the linear range for each gene. β-actin mRNA was amplified as an internal control by RT-PCR using specific primers. Amplified products were analyzed by electrophoresis on 1.2% agarose gels for galectin-7 and β-actin or 1.8% gels for Egr-1 using Sybr Safe staining and UV illumination.

### Generation of Stable Transfectants Expressing Luciferase and Galectin-7

To obtain stable B16F1-Luc transfectants expressing the *luciferase* reporter gene under the control of the SV40 promoter, B16F1 cells were co-transfected with linearized pGL3-(SV40) vector (Promega) and the pSrα vector that conferred puromycin resistance. After 48 h of culture in complete medium, transfected cells were grown in complete medium containing 2 µg/mL of puromycin before individual colonies were selected and expanded. Clones expressing constitutively high levels of luciferase (clones #1 and #9) were then used to generate stable transfectants expressing constitutively high levels of galectin-7. For this purpose, we used the pRc-CMV2-galectin-7 vector encoding the murine *galectin-7* gene (GenBank accession no. AF 331640). Individual colonies were selected, expanded and assayed for galectin-7 expression by RT-PCR, ELISA and confocal microscopy. Control transfectants were generated using the empty pSrα vector, as described [20].

### Proliferation Assay

Three clones of B16F1 cells overexpressing galectin-7 (B16F1-G7 #5, 10 and 14) and three control cell lines (B16F1-srα #1, 2 and 3) were seeded at 2×10^3^ cells/well in 96-well culture plates. Once the cells were confluent, quercetin was added at 10 µg/mL (dissolved in DMSO) for 72 h. In control wells, 1% DMSO solution was added. DNA synthesis was assayed by adding 1 µCi of methyl-[^3^H]thymidine/well and incubating the cells for 16 h. Radioactivity was measured after adding a scintillation cocktail using a scintillation counter (Trilux, 1450 microbeta, Wallac). The experiment was performed in triplicate and repeated three times.

### Luciferase Assay

Luciferase activity in cell lines and tissues was measured as previously described [28]. Briefly, for *ex vivo* imaging, mice were injected i.p. with 150 mg/kg of D-luciferin. Ten minutes later, the mice were sacrificed, and lungs were collected. After imaging, lungs were homogenized in cell culture lysis reagent (CCLR), snap frozen in liquid nitrogen and thawed at 37°C before luciferase assays. For cell lines, B16F1 transfectant cells (10^6^ cells) were lysed in 100 µL of CCLR containing phenylmethylsulfoxide at 4°C for 1 h and then vortexed for 30 sec. After centrifugation for 20 min at 4°C, the protein concentration of the supernatant was measured by the Bradford method. Equal amounts of protein were used to determine luciferase activity. Luciferase activity was measured using the Luciferase Assay System and a Lumat LN 9507 Luminometer (Berthold Technologies, Oak Ridge, TN).

### Tumorigenic and Metastatic Assays

Male or female C57BL/6 mice and galectin-7-deficient mice (KOG7) (6 to 8 weeks old) were injected subcutaneously (s.c.) into the left flank with 5×10^4^ B16F1 cells. Animals were monitored periodically for tumor growth, which did not exceed a volume of 2500 mm^3^. The length (L) and width (W) of the tumor were measured using calipers fitted with a vernier scale, and the size of the tumor was calculated using the formula L^2^×W×0.4. When the maximum tumor volume was reached, mice were sacrificed, and the tumors were divided and frozen for PCR analysis or fixed in a buffered formaldehyde solution for immunohistochemistry (IHC). To induce the dissemination of B16F1 melanoma cells in the lung, male or female C57BL/6 mice (6 to 8 weeks old) were injected in the tail vein with 2×10^5^ B16F1 luciferase transfectant cells that were either control or overexpressed galectin-7. Animals were monitored periodically for clinical signs of tumor growth. When moribund or at a specific time, the mice were sacrificed, and lungs were collected and examined by *ex vivo* imaging. Tissues were homogenized in CCLR for luciferase assays as described above.

### Immunohistochemistry

Primary and metastatic lung tumors were fixed and processed for IHC analysis as described previously [22]. Briefly, 3-µm-thick sections were prepared from each tissue sample. Immunostaining reactions for galectin-7 were carried out using the Discovery XT automated immunostainer (Ventana Medical Systems, Tucson, AZ). Deparaffinized sections were incubated in cell conditioning solution (pH 8.0) for antigen retrieval and then stained for 60 min with an anti-human galectin-7 monoclonal antibody at a 1∶150 dilution. The slides were counterstained with hematoxylin and bicarbonate. Each section was scanned at a high resolution (Nanozoomer, Hammamatsu Photonics K.K). A total of 13 human malignant melanomas and 47 nevus were analyszed from samples obtained with a written informed consent and with the approval of the research ethics committee of the research center at the Centre Hospitalier de l’Université de Montréal.

### ELISA Assays

Cells were lysed with RIPA lysis buffer at 4°C for 1 h and then centrifuged at 12000 rpm for 20 min at 4°C. Protein concentrations were measured by the Bradford method. Amounts of 100 or 200 µg of total protein per well were analyzed using the murine galectin-7 ELISA kit (R & D Systems) according to the manufacturer’s instructions. For supernatant analysis, aliquots of 4×10^4^, 1.3×10^3^ and 450 cells were plated in 96 wells plate in 100 µL of culture media and incubated for 24, 48 and 72 h respectively to obtain 100% confluent cells in each well. The total volume of supernatant was used for the ELISA assay.

### Motility Assay

Confluent cultures grown in six-well culture plates were wounded with a 200 µL pipette tip (time 0) and incubated for 16 h. The wells were inspected with an inverted light microscope (Nikon eclipse TE2000-U) using a 10X objective. Images were captured using a coolSNAP HQ camera and analyzed using the Metamorph software (Universal Imaging Corporation).

### Transient Transfection

B16F1 cells and cells from a mix of three B16F1-G7 clones (G7#5, #10, and #14) were plated at a concentration of 10^5^ cells per well in six-well plates and incubated at 37°C overnight. Thereafter, cells were co-transfected with 0.2 µg of PGL3-EGR-1 reporter construct and 0.2 µg of pRLSV40-Renilla vector, for transfection control, using 5 µL of Lipofectamine 2000 reagent according to the manufacturer’s protocol (Invitrogen). After 24 h, transfected cells were treated with increasing concentrations of quercetin (0–100 µg/mL) for 24 h. Cells were lysed with 100 µL of passive lysis buffer containing PMSF at 4°C for 1 h and centrifuged for 20 min at 4°C. Equal amounts of protein were analyzed by the Dual-Luciferase Assay system according to the manufacturer’s instructions (Promega).

### Statistical Data Analysis

Data are presented as means ± SD. Student *t* test was used to test for statistical significance that was established at *p*<0.05.

## Results

### Galectin-7 Expression in Human Melanoma Tissues

**Figure 1:**
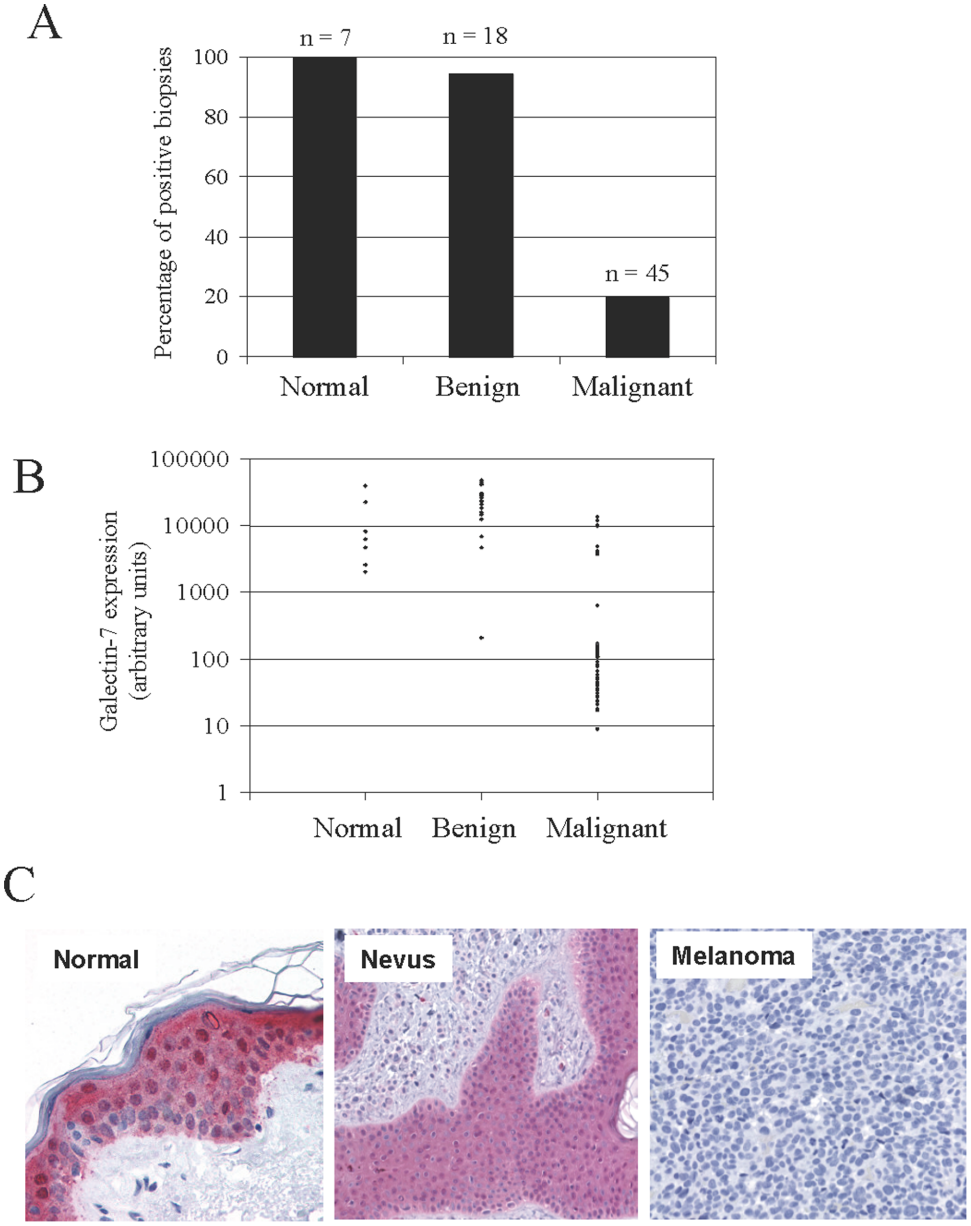
Galectin-7 expression in human melanoma tissues. A) Percentage of positive biopsies of normal skin (n = 7), nevus (n = 18) and malignant melanoma (n = 45), as determined by the *in silico* analysis of galectin-7 expression from a microarray of human biopsies [26]. B) Representative graph of galectin-7 mRNA expression in each biopsy described in (A). C) Detection of galectin-7 by immunohistochemistry in representative biopsies of nevi and malignant melanoma. Overall, 13 malignant melanomas and 47 nevi were tested.

**Figure 2:**
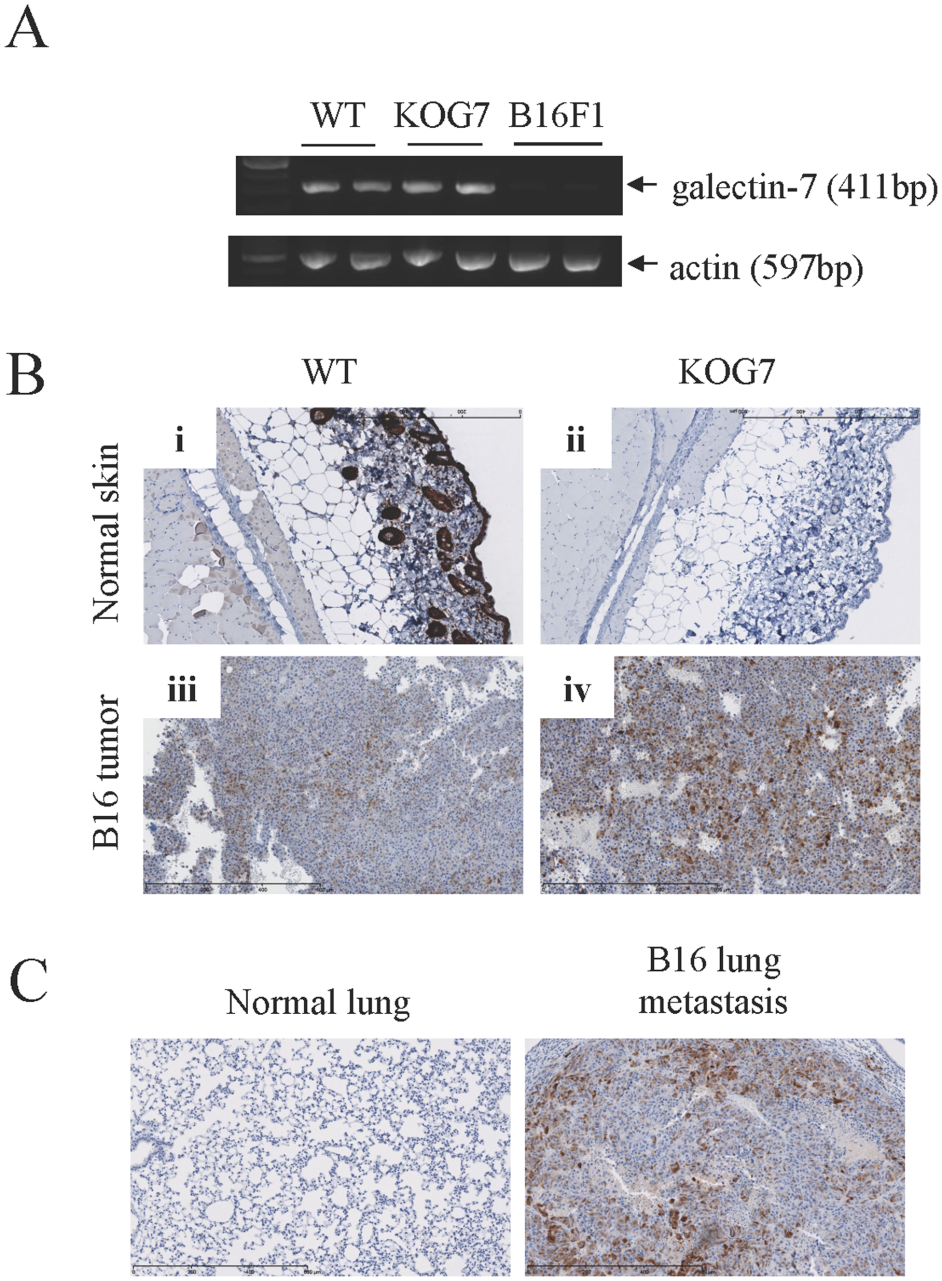
Increased galectin-7 expression in B16F1 primary tumors and lung metastases. Primary tumors were collected at necropsy 18 days after the subcutaneous injection of B16F1 cells (5×10^4^ cells) in C57BL/6 (WT) and galectin-7-deficient mice (KOG7). (A) RT-PCR analysis of galectin-7 mRNA expression in two B16F1 primary tumors from WT and KOG7 mice in comparison with the B16F1 cell line. Actin was used as a loading and specificity control. B) Immunohistochemistry for galectin-7 in normal skin (i, ii) and B16F1 primary tumors (iii, iv) in WT and KOG7 mice. These galectin-7-positive structures located in the suprabasal epidermis have been reported before and likely represent suprabasal keratinocytes, which are known to express galectin-7 constitutively [13]. Control stained without HRP and in absence of Abs did not show any detectable staining in both wt and KOG7 mice. C) Immunohistochemistry for galectin-7 in a lung collected 20 days post-injection from one KOG7 mouse injected intravenously via the tail vein with B16F1 cells (2×10^5^ cells) in comparison with a normal lung. Scale bars in all immunohistochemistry images represent 600 µM.

We first conducted an *in silico* analysis of galectin-7 expression using datasets from the Gene Expression Omnibus (GEO) repository of the National Center for Biotechnology Information (NCBI). For this purpose, we examined a dataset of microarray data obtained from the profiling of 45 malignant melanomas and 18 nevi (GDS1375) [29]. These data showed that galectin-7 was detected at the mRNA level in all biopsies of normal skin (n = 7) and in most biopsies collected from patients with nevi (17/18) (**Fig. 1a**). In biopsies obtained from patients with malignant melanomas, galectin-7 expression was relatively rare (9/45) (**Fig. 1b**). Similar expression patterns were also observed for benign versus malignant melanoma in a dataset collected from the whole-genome profiling of the melanoma progression pathway (GDS1989) [30]. Low expression levels of galectin-7 were also observed in both human and mouse malignant melanoma cell lines [31] (**Fig. 1c** and **Figure S1**). Interestingly, however, we found that galectin-7 can be upregulated in B16 melanoma cells that had been injected subcutaneously into normal C57BL/6 syngeneic mice (**Fig. 2a**). IHC staining of B16 tumors in galectin-7-deficient (KOG7) mice confirmed that galectin-7 expression in the B16 tumors was not due to expression by surrounding stromal cells (**Fig. 2b**). Interestingly, a stronger galectin-7 staining was observed in KOG7 mice as compared to WT mice. The reason for such a difference is unclear at present. This upregulation of galectin-7 in B16 melanoma cells *in vivo* was also observed in lung biopsies of KOG7 mice injected i.v. with B16 cells (**Fig. 2c**).

### Generation of a B16 Melanoma Cell Line Constitutively Expressing Galectin-7

**Figure 3:**
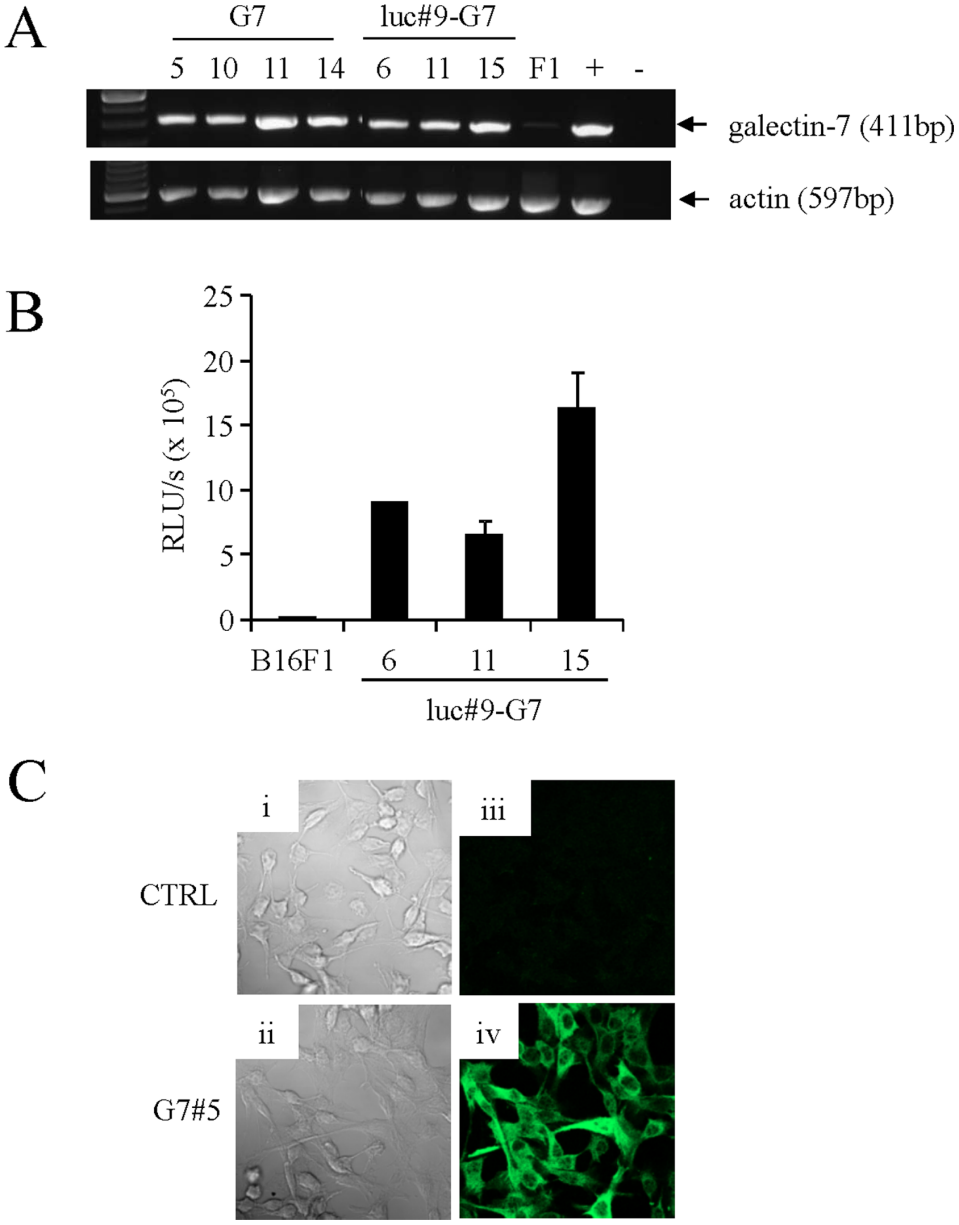
Validation of B16F1 transfectants overexpressing luciferase and/or galectin-7. A) RT-PCR analysis for galectin-7 mRNA expression in transfectant cells overexpressing galectin-7 with or without luciferase in comparison with a control B16F1 cell line (F1). The aggressive murine lymphoma cell line S19 (+) was used as a positive control. Actin was used as a loading and specificity control. B) Luciferase assay of three B16F1 transfectant cell lines overexpressing luciferase and cotransfected with pRc-CMV2-galectin-7 in comparison with the control B16F1 cell line. C) Confocal microscopy for galectin-7 in control B16F1 cells (iii) and galectin-7 transfectant cells (G7#5) (iv); these cells were also visualized (i, ii).

Because overexpression of galectin-7 is known to regulate tumor growth and metastasis in multiple tumor cell types, we next investigated whether galectin-7 could modulate the growth of primary tumors and the dissemination of metastasis. For this purpose, we generated a series of stable B16 transfectants constitutively expressing galectin-7 at both the mRNA and protein levels (**Fig. 3**). The transfectants were also co-transfected with an expression vector encoding the *firefly luciferase* gene to facilitate *in vivo* follow-up of metastases to the lung. As previously reported, galectin-7 expression was restricted to the intracellular compartment and was not detected in the cultured supernatant of B16 transfectants (**Fig. 3c** and **Figure S2**).

### Galectin-7 Reduces the Motility, Induces Resistance to Apoptosis and Increases egr-1 (Early Growth Response Protein 1) in B16 Melanoma Cells

**Figure 4:**
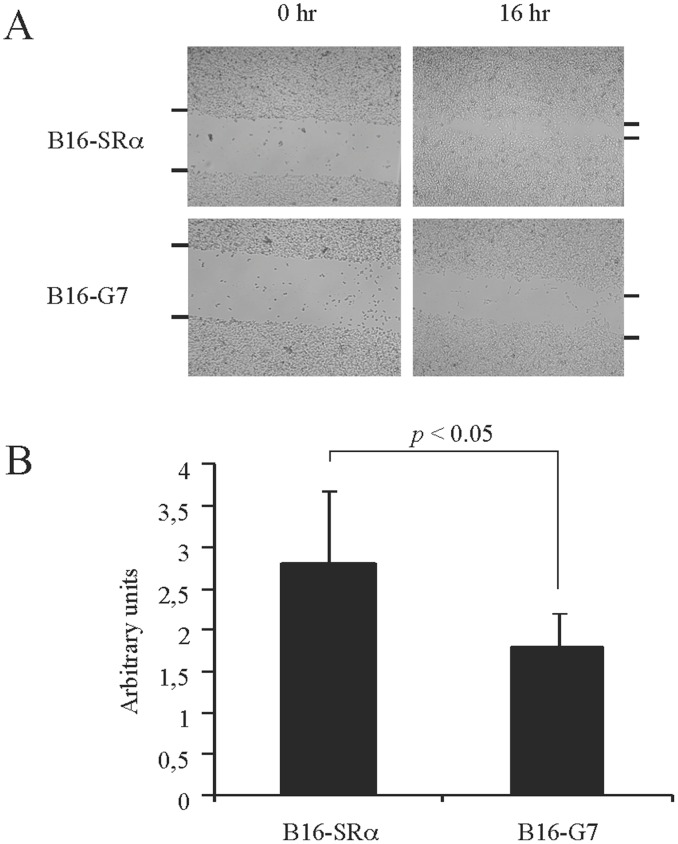
Effect of galectin-7 on B16F1 cell migration. A) Images of the motility assay after the wounding of confluent B16F1 cells overexpressing galectin-7 (B16-G7) or controls (B16-Srα) at T = 0 and T = 16 hr. A mixture of three clones of B16F1-G7 was used (G7 #5, 10 and 14). B) Representative graph of the results obtained in (A).

**Figure 5:**
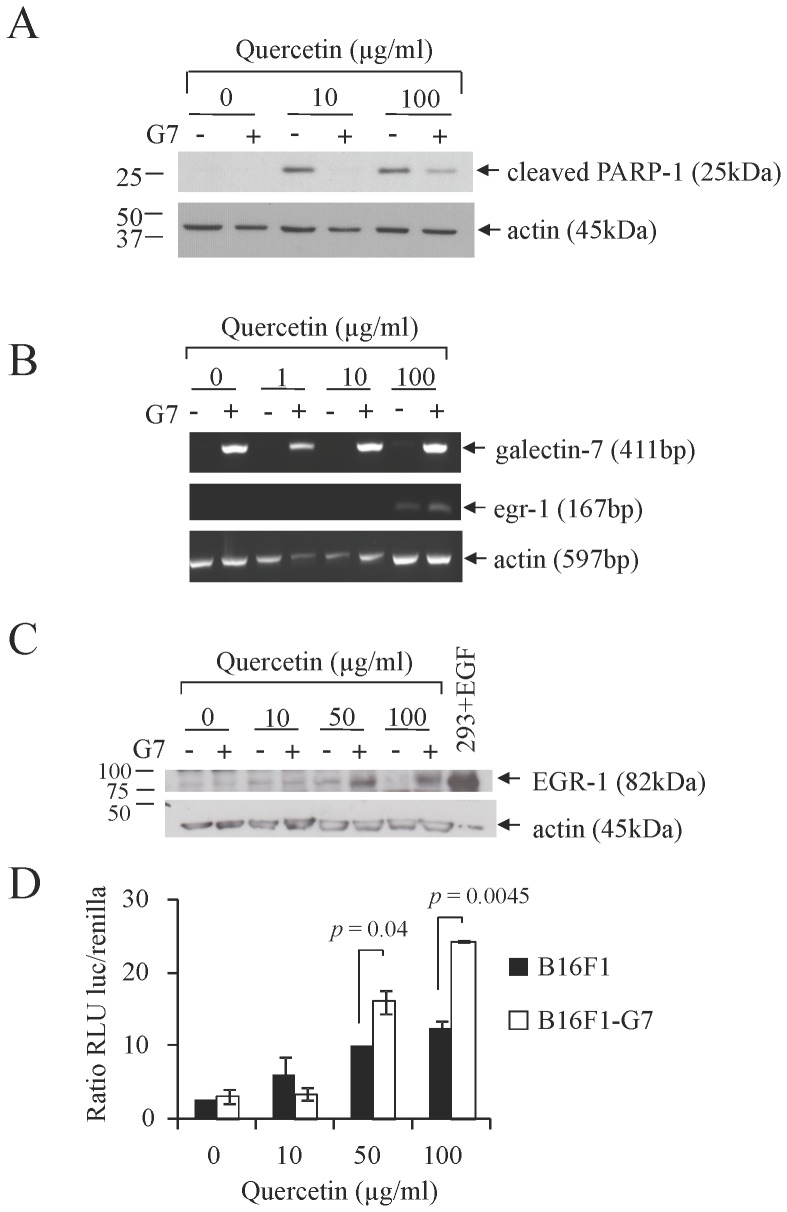
Effect of quercetin on B16F1 cells overexpressing galectin-7 on apoptosis and EGR-1 expression. B16F1 cells overexpressing galectin-7 (+) or controls (–) were treated with various doses of quercetin. A) Apoptotic sensitivity was analyzed by western blotting for cleaved PARP-1 detection. B) RT-PCR analysis of galectin-7 and EGR-1 mRNA expression. C) Western blot analysis for EGR-1 detection, 293 cells transfected with EGF were used as a positive control. Actin was used as a loading and specificity control. D) Dual luciferase assay of B16F1 transfectant cells overexpressing galectin-7 (□) or controls (▪) co-transfected with luciferase reporter plasmids with an EGR-1 promoter and pRLSV40-Renilla vector as a transfection control and treated with various doses of quercetin for 24 h. A mixture of three clones of B16F1-G7 was used (G7 #5, 10 and 14) for all of these experiments.

The expression of galectin-7 in B16 cells significantly reduced their cellular motility (**Fig. 4**). While galectin-7 did not affect the *in vitro* proliferation rate of the cells, it did inhibit their sensitivity to apoptosis induced by quercetin in a dose-dependent manner (**Fig. 5** **and Figure S3**). This effect of galectin-7 on quercetin-induced apoptosis was concomitant with its ability to upregulate both the mRNA and protein levels of EGR-1, consistent with previous results obtained in human colon carcinoma cell line [32]. EGR-1 is a master regulator that plays an important role in a variety of cellular processes in cancer cells [33]. The ability of galectin-7 to increase EGR-1 at the transcriptional level in B16F1 cells was confirmed using a reporter vector encoding the *egr-1* promoter (**Fig. 5d**).

### In vivo Growth of B16 Cells Expressing High Levels of Galectin-7

**Figure 6:**
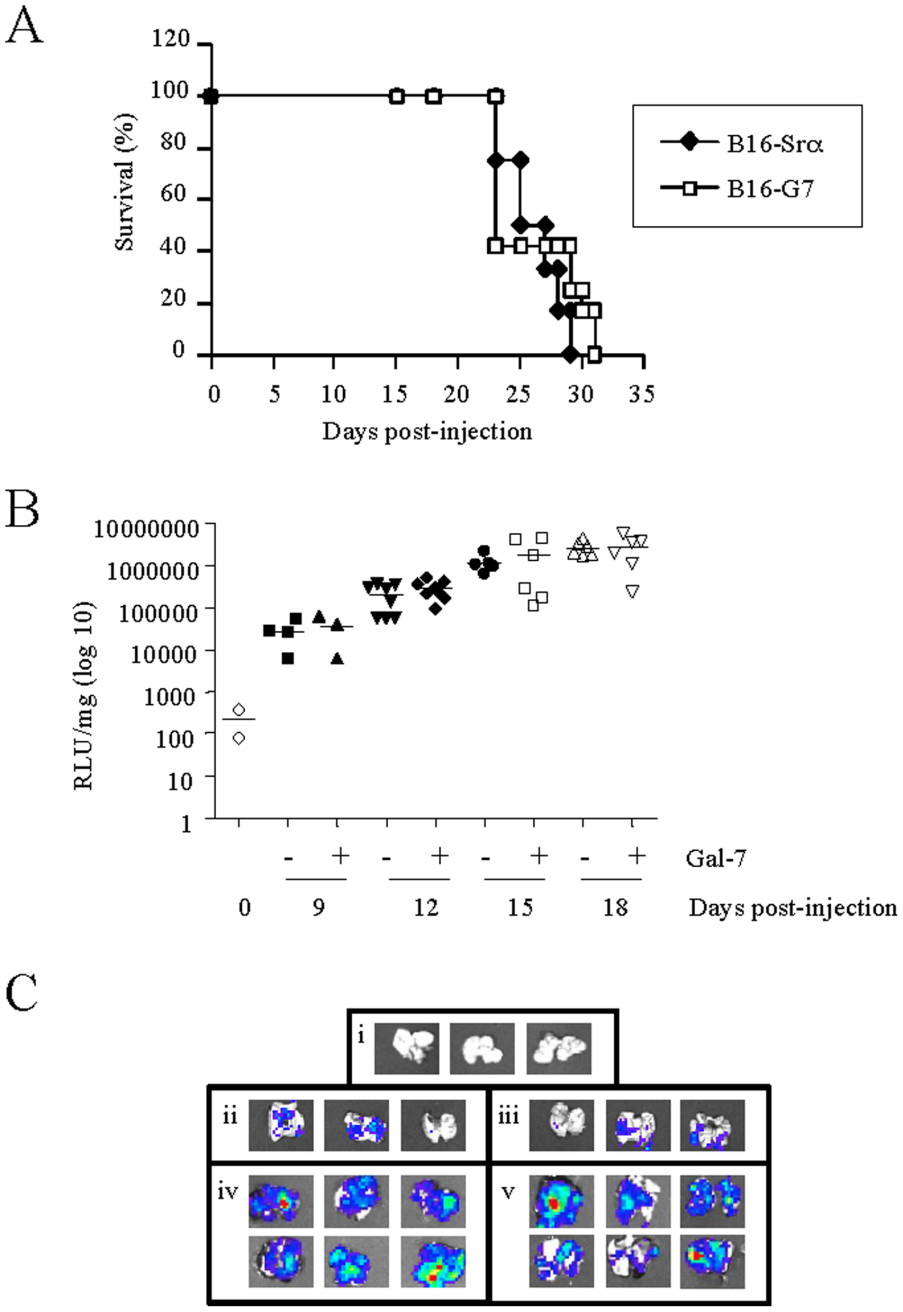
Effect of galectin-7 in B16F1 cells on survival and metastasis in lungs. A) Survival curve of C57BL/6 mice injected i.v. with B16F1 transfectant cells overexpressing galectin-7 (□) or controls (♦) (2×10^5^ cells) (n = 8−10). B) Luciferase assay of lungs of C57BL/6 mice sacrificed at 9, 12, 15 or 18 days after the i.v. injection of a mixture of B16F1 luciferase transfectant cells overexpressing galectin-7 (+) or control (–) (n = 3−8). Normal lungs were used as a negative control (T: 0; n = 2). C) *Ex vivo* imaging of a lung metastasis, as seen in (B), of B16F1 cells overexpressing galectin-7 (iii and v) or control cells (ii and iv) at 9 and 18 days in comparison with control lungs (i).

We next investigated whether galectin-7 could modulate the tumor progression of B16 melanoma cells. For this purpose, we first compared the growth of B16 cells expressing high levels of galectin-7 (B16-G7) with control cells (B16-Srα). Our results showed that mice injected with B16 cells had similar survival rates regardless of galectin-7 expression (26±2.3 days (n = 12) for galectin-7-expressing cells vs. 25.9±3.7 days (n = 12) for controls) (**Fig. 6a**). This result was observed in independent experiments using either 1×10^4^ or 5×10^4^ cells (*data not shown*). To study the effect of galectin-7 on melanoma metastasis, we used genetically engineered B16F1 cells overexpressing luciferase. This cell line model facilitates cancer cell detection in target organs with a luciferase assay or the *ex vivo* bioluminescence imaging of organs. Although we observed a time-dependent increase in the metastatic load in the lung, the number of metastases was similar in mice injected with galectin-7 transfectant cells compared with those injected with control cells (**Fig. 6b and 6c**).

## Discussion

Galectin-7 is normally expressed in stratified epithelia, most notably in the skin epidermis. In cancer, its expression is often altered, although its role in cancer biology is debated. A number of indications have suggested that galectin-7 may potentially be important in melanoma proliferation, invasion, and metastasis. Like other members of the galectin family, galectin-7 has been shown to either positively or negatively modulate apoptosis and tumor growth [34]. In colon cancer, for example, galectin-7 has an anti-tumorigenic function [15], in contrast to its pro-tumorigenic function reported in lymphoma, breast cancer, squamous cell carcinoma of the tongue and esophagus, and thyroid malignancies [20]-[22], [35]–[37]. In skin cancer, a recent study has shown that galectin-7 is induced during neoplastic transformation of the skin following exposure to cypermethrin, a highly carcinogenic insecticide used for agricultural and domestic applications [38]. Because melanoma represents one of the most dangerous forms of skin cancer, we investigated the role of galectin-7 in melanoma. In the present work, we have shown that 1) galectin-7 is rarely expressed in biopsies of malignant melanoma, 2) galectin-7 reduces the motility of B16F1 cells, and 3) galectin-7 increases the resistance of B16F1 cells to apoptosis and the expression of EGR-1. We also found that overexpression of galectin-7 is insufficient to modulate the growth of primary tumors or the dissemination of B16 melanoma cells to the lung.

Our data showing that galectin-7 reduces the motility of melanoma cells are novel and worth investigating further in the context of the ability of Bcl-2 to bind galectin-7 [39]. Although the functional relevance of such an interaction is likely to explain the ability of galectin-7 to modulate apoptosis, it could also be important for cell motility because as suggested by a recent study showing that cytoplasmic Bcl-2 inhibits cell motility and enhances F-actin polymerization during cell spreading [40]. Moreover, Villeneuve *et al*. [39] have shown that in keratinocytes, galectin-7 colocalizes with cortactin, an actin-binding protein implicated in membrane ruffle formation. It is noteworthy, however, that reduced *in vitro* motility did not affect the number of metastases to the lung, suggesting caution when assigning correlations between *in vitro* and *in vivo* observations. Although cell motility and/or EGR-1 are likely to be important in tumor progression, it is logical to assume that their relative importance is tumor and context dependent.

*In silico* analysis of public datasets has shown that galectin-7 mRNA is detected in most if not all nevi. Our IHC data and the fact that galectin-7 is constitutively expressed at high levels in skin epidermis suggest that such signal likely represents that presence of epithelial cells in the biopsies of nevi. Such explanation may also be true for malignant melanoma. These results emphasize the risk of surrounding tissue contamination when performing analysis of biopsies. Nevertheless, our results showing that B16 cells do express galectin-7 when transplanted *in vivo* leaves open the possibility that in rare cases, galectin-7 could be expressed in malignant melanoma cells. Future IHC analysis on normal melanocytes and in a larger number of biopsies of nevus and melanoma are needed to determine the utility of galectin-7 as a predictive biomarker in melanom. Our *in vivo* results using stable transfectants overexpressing galectin-7 suggest, however, that if indeed galectin-7 can be found in rare cases of melanoma, it probably plays a very limited role in tumor growth and apoptosis. The importance of galectin-7 in melanoma is thus very distinct from what has been observed in other types of epithelial cancer.

## Supporting Information

Figure S1 **Expression of galectin-7 in murine and human melanoma cell lines.** Galectin-7 expression in the aggressive variant B16F10 murine melanoma cells in comparison to the parental cell line B16F1 analyzed by A) RT-PCR for galectin-7 mRNA expression (Actin was used as loading and specificity control) and B) ELISA assay for galectin-7 concentration in comparison with 164T2 non aggressive lymphoma cells and its aggressive variant S19. C) ELISA assay for galectin-7 concentration in human melanoma cell lines (888mel, 537mel, SK23 and Mel-FB) in comparison to MDA-MB-468 breast cancer cell line as positive control.
(TIFF)

Figure S2 **ELISA assay for galectin-7 concentration in culture supernatant of B16F1 tranfectant cells overexpressing (□) or not (▪) galectin-7 after 24, 48 and 72 hours of incubation.** Lysed cells from this experiment were used as positive control.
(TIFF)

Figure S3 **Cellular proliferation was analyzed by thymidine incorporation after 72 hours of quercetin (10 µg/ml) treatment in B16F1 transfectant cells overexpressing (□) or not (▪) galectin-7.** Results are representative of three independent experiments.
(TIFF)

## References

[1] Miller AJ, Mihm MC Jr (2006) Melanoma. N Engl J Med 355: 51–65. 16822996 10.1056/NEJMra052166

[2] Ivanov VN, Bhoumik A, Ronai Z (2003) Death receptors and melanoma resistance to apoptosis. Oncogene 22: 3152–3161. 12789291 10.1038/sj.onc.1206456

[3] Hersey P (2006) Apoptosis and melanoma: how new insights are effecting the development of new therapies for melanoma. Curr Opin Oncol 18: 189–196. 16462190 10.1097/01.cco.0000208794.24228.9f

[4] Sapoznik S, Hammer O, Ortenberg R, Besser MJ, Ben-Moshe T, et al.. (2012) Novel anti-melanoma immunotherapies: disarming tumor escape mechanisms. Clin Dev Immunol. : Apr 23. 10.1155/2012/818214 PMC3386565 22778766

[5] Barondes SH, Cooper DN, Gitt MA, Leffler H (1994) Galectins. Structure and function of a large family of animal lectins. J Biol Chem 269: 20807–20810. 8063692

[6] Elola MT, Wolfenstein-Todel C, Troncoso MF, Vasta GR, Rabinovich GA (2007) Galectins: matricellular glycan-binding proteins linking cell adhesion, migration, and survival. Cell Mol Life Sci 64: 1679–1700. 17497244 10.1007/s00018-007-7044-8 PMC11136145

[7] Brewer CF, Miceli MC, Baum LG (2002) Clusters, bundles, arrays and lattices: novel mechanisms for lectin-saccharide-mediated cellular interactions. Curr Opin Struct Biol 12: 616–23. 12464313 10.1016/s0959-440x(02)00364-0

[8] Wang JL, Gray RM, Haudek KC, Patterson RJ (2004) Nucleocytoplasmic lectins. Biochim Biophys Acta 1673: 75–93. 15238251 10.1016/j.bbagen.2004.03.013

[9] Liu FT, Rabinovich GA (2005) Galectins as modulators of tumour progression. Nat Rev Cancer 5: 29–41. 15630413 10.1038/nrc1527

[10] Delacour D, Koch A, Jacob R (2009) The role of galectins in protein trafficking. Traffic 10: 1405–13. 19650851 10.1111/j.1600-0854.2009.00960.x

[11] Rabinovich GA, Toscano MA (2009) Turning ‘sweet’ on immunity: galectin-glycan interactions in immune tolerance and inflammation. Nat Rev Immunol 9: 338–52. 19365409 10.1038/nri2536

[12] Madsen P, Rasmussen HH, Flint T, Gromov P, Kruse TA, et al (1995) Cloning, expression, and chromosome mapping of human galectin-7. J Biol Chem 270: 5823–5829. 7534301 10.1074/jbc.270.11.5823

[13] Magnaldo T, Bernerd F, Darmon M (1995) Galectin-7, a human 14-kDa S-lectin, specifically expressed in keratinocytes and sensitive to retinoic acid. Dev Biol 168: 259–271. 7729568 10.1006/dbio.1995.1078

[14] Magnaldo T, Fowlis D, Darmon M (1998) Galectin-7, a marker of all types of stratified epithelia. Differentiation 63: 159–168. 9697310 10.1046/j.1432-0436.1998.6330159.x

[15] Ueda S, Kuwabara I, Liu FT (2004) Suppression of tumor growth by galectin-7 gene transfer. Cancer Res 64: 5672–5676. 15313906 10.1158/0008-5472.CAN-04-0985

[16] Polyak K, Xia Y, Zweier JL, Kinzler KW, Vogelstein B (1997) A model for p53-induced apoptosis. Nature 389: 300–305. 9305847 10.1038/38525

[17] Zhu H, Pei HP, Zeng S, Chen J, Shen LF, et al (2009) Profiling protein markers associated with the sensitivity to concurrent chemoradiotherapy in human cervical carcinoma. J Proteome Res 8: 3969–3976. 19507834 10.1021/pr900287a

[18] Lu J, Pei H, Kaeck M, Thompson HJ (1997) Gene expression changes associated with chemically induced rat mammary carcinogenesis. Mol Carcinog 20: 204–215. 9364210 10.1002/(sici)1098-2744(199710)20:2<204::aid-mc7>3.0.co;2-m

[19] Saussez S, Cucu DR, Decaestecker C, Chevalier D, Kaltner H, et al (2006) Galectin 7 (p53-Induced Gene 1): A New Prognostic Predictor of Recurrence and Survival in Stage IV Hypopharyngeal Cancer. Ann Surg Oncol 13: 999–1009. 16788763 10.1245/ASO.2006.08.033

[20] Demers M, Magnaldo T, St-Pierre Y (2005) A novel function for galectin-7: promoting tumorigenesis by up-regulating MMP-9 gene expression. Cancer Res 65: 5205–5210. 15958565 10.1158/0008-5472.CAN-05-0134

[21] Demers M, Biron-Pain K, Hebert J, Lamarre A, Magnaldo T, et al (2007) Galectin-7 in lymphoma: elevated expression in human lymphoid malignancies and decreased lymphoma dissemination by antisense strategies in experimental model. Cancer Res 67: 2824–2829. 17363605 10.1158/0008-5472.CAN-06-3891

[22] Demers M, Rose AA, Grosset AA, Biron-Pain K, Gaboury L, et al (2010) Overexpression of galectin-7, a myoepithelial cell marker, enhances spontaneous metastasis of breast cancer cells. Am J Pathol 176: 3023–3031. 20382700 10.2353/ajpath.2010.090876 PMC2877862

[23] Nakahara S, Oka N, Raz A (2005) On the role of galectin-3 in cancer apoptosis. Apoptosis 10: 267–275. 15843888 10.1007/s10495-005-0801-y

[24] Gendronneau G, Sidhu SS, Delacour D, Dang T, Calonne C, et al (2008) Galectin-7 in the control of epidermal homeostasis after injury. Mol Biol Cell 19: 5541–5549. 18829868 10.1091/mbc.E08-02-0166 PMC2592666

[25] Fidler IJ (1975) Biological behavior of malignant melanoma cells correlated to their survival in vivo. Cancer Res 35: 218–224. 1109790

[26] Robbins PF, El-Gamil M, Li YF, Zeng G, Dudley M, et al (2002) Multiple HLA class II-restricted melanocyte differentiation antigens are recognized by tumor-infiltrating lymphocytes from a patient with melanoma. J Immunol 169: 6036–6047. 12421991 10.4049/jimmunol.169.10.6036 PMC2410044

[27] Aoudjit F, Potworowski EF, St-Pierre Y (1998) The metastatic characteristics of murine lymphoma cell lines in vivo are manifested after target organ invasion. Blood 91: 623–629. 9427718

[28] Biron-Pain K, St-Pierre Y (2012) Monitoring mmp-9 gene expression in stromal cells using a novel transgenic mouse model. Cell Mol Life Sci 69: 783–91. 21833585 10.1007/s00018-011-0777-4 PMC11114778

[29] Talantov D, Mazumder A, Yu JX, Briggs T, Jiang Y, et al (2005) Novel genes associated with malignant melanoma but not benign melanocytic lesions. Clin Cancer Res 11: 7234–7242. 16243793 10.1158/1078-0432.CCR-05-0683

[30] Smith AP, Hoek K, Becker D (2005) Whole-genome expression profiling of the melanoma progression pathway reveals marked molecular differences between nevi/melanoma in situ and advanced-stage melanomas. Cancer Biol Ther 4: 1018–1029. 16251803 10.4161/cbt.4.9.2165

[31] Okamoto I, Pirker C, Bilban M, Berger W, Losert D, et al (2005) Seven novel and stable translocations associated with oncogenic gene expression in malignant melanoma. Neoplasia 7: 303–311. 15967107 10.1593/neo.04514 PMC1501156

[32] Lim J H, Park J W, Min DS, Chang J S, Lee YH, et al (2007) NAG-1 up-regulation mediated by EGR-1 and p53 is critical for quercetin-induced apoptosis in HCT116 colon carcinoma cells. Apoptosis 12: 411–421. 17191121 10.1007/s10495-006-0576-9

[33] Ahmed MM (2004) Regulation of radiation-induced apoptosis by early growth response-1 gene in solid tumors. Curr Cancer Drug Targets 4: 43–52. 14965266 10.2174/1568009043481704

[34] St-Pierre Y, Campion CG, Grosset AA (2012) A distinctive role for galectin-7 in cancer? Front Biosci 17: 438–450. 10.2741/3937 22201754

[35] Rorive S, Eddafali B, Fernandez S, Decaestecker C, Andre S, et al (2002) Changes in galectin-7 and cytokeratin-19 expression during the progression of malignancy in thyroid tumors: diagnostic and biological implications. Mod Pathol 15: 1294–1301. 12481010 10.1097/01.MP.0000037306.19083.28

[36] Zhu X, Ding M, Yu ML, Feng MX, Tan LJ, et al (2010) Identification of galectin-7 as a potential biomarker for esophageal squamous cell carcinoma by proteomic analysis. BMC Cancer 10: 290. 20546628 10.1186/1471-2407-10-290 PMC3087317

[37] Alves PM, Godoy GP, Gomes DQ, Medeiros AM, de Souza LB, et al (2011) Significance of galectins-1, -3, -4 and -7 in the progression of squamous cell carcinoma of the tongue. Pathol Res Pract 207: 236–240. 21397408 10.1016/j.prp.2011.02.004

[38] George J, Srivastava AK, Singh R, Shukla Y (2011) Cypermethrin exposure leads to regulation of proteins expression involved in neoplastic transformation in mouse skin. Proteomics 11: 4411–4421. 21919204 10.1002/pmic.201100233

[39] Villeneuve C, Baricault L, Canelle L, Barboule N, Racca C, et al (2011) Mitochondrial proteomic approach reveals galectin-7 as a novel BCL-2 binding protein in human cells. Mol Biol Cell 22: 999–1013. 21289092 10.1091/mbc.E10-06-0534 PMC3069024

[40] Ke H, Parron VI, Reece J, Zhang JY, Akiyama SK, et al (2010) BCL2 inhibits cell adhesion, spreading, and motility by enhancing actin polymerization. Cell Res 20: 458–469. 20142842 10.1038/cr.2010.21 PMC2848692

