# Supplementary figures and images for: Expression and Functions of Galectin-7 in Human and Murine Melanomas
Source: PLoS One. 2013 May 3;8(5):e63307. doi: 10.1371/journal.pone.0063307 (PMC3643947; doi:10.1371/journal.pone.0063307)

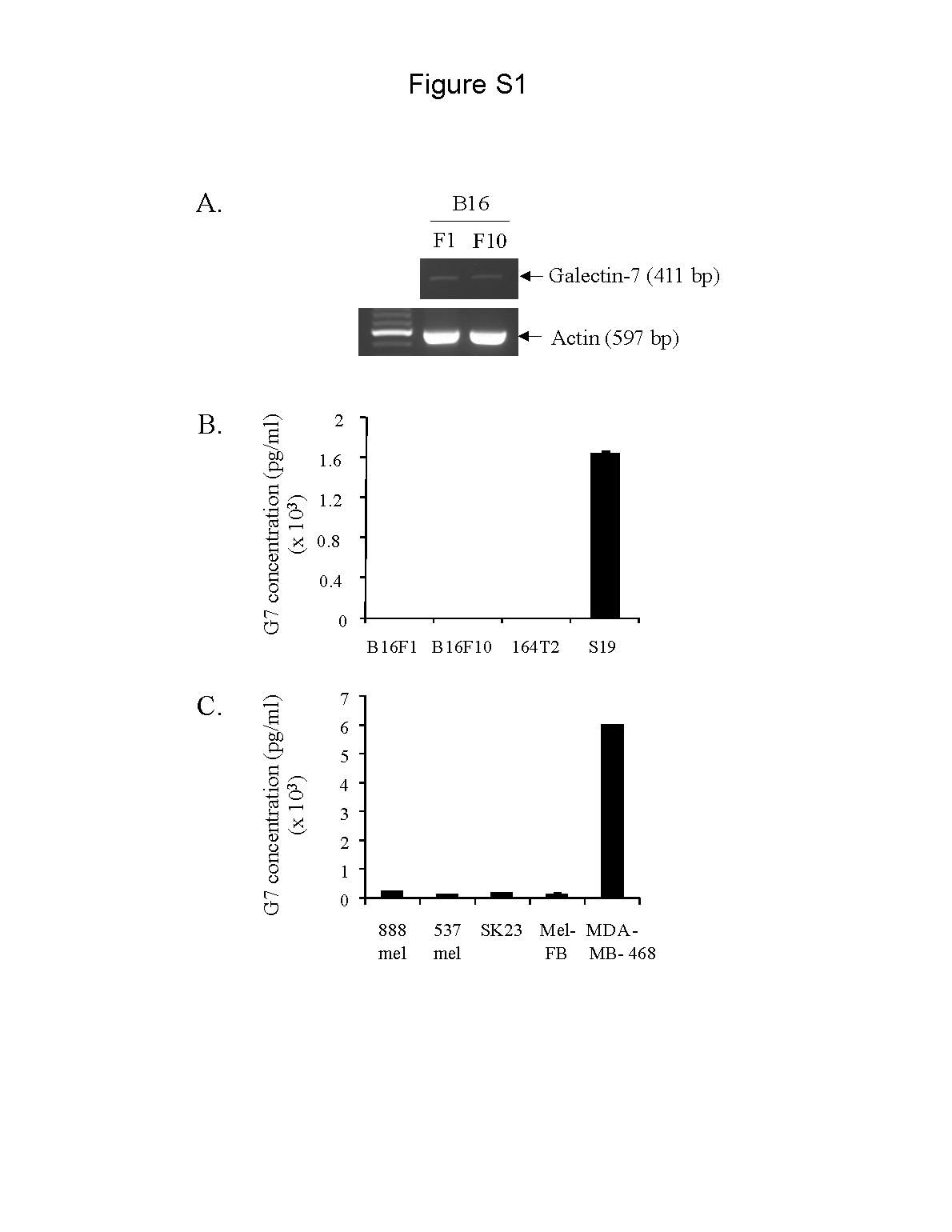

Supplement: Figure S1 — Expression of galectin-7 in murine and human melanoma cell lines. Galectin-7 expression in the aggressive variant B16F10 murine melanoma cells in comparison to the parental cell line B16F1 analyzed by A) RT-PCR for galectin-7 mRNA expression (Actin was used as loading and specificity control) and B) ELISA assay for galectin-7 concentration in comparison with 164T2 non aggressive lymphoma cells and its aggressive variant S19. C) ELISA assay for galectin-7 concentration in human melanoma cell lines (888mel, 537mel, SK23 and Mel-FB) in comparison to MDA-MB-468 breast cancer cell line as positive control. (TIFF) [file pone.0063307.s001.tiff]
